# 2015 Behavioral clinical trials: Considerations for design and conduct using the new NIH study protocol template

**DOI:** 10.1017/cts.2018.197

**Published:** 2018-11-21

**Authors:** Susan L. Murphy, Nancy Yovetich, Melissa Riddle

**Affiliations:** University of Michigan School of Medicine

## Abstract

OBJECTIVES/SPECIFIC AIMS: (1) To discuss key differences of behavioral clinical trials from trials involving drugs, devices, and biologics and (2) to discuss NIH efforts to provide a study protocol template for use by investigators conducting behavioral clinical trials. METHODS/STUDY POPULATION: A working group was convened by NIH to refine the commonly used protocol template required for investigators conducting Phase 2 or 3 NIH-funded clinical trials. The committee met by phone regularly for 4 months to review, discuss, and refine each section of the template as needed to include aspects relevant to behavioral trials. RESULTS/ANTICIPATED RESULTS: The behavioral trial protocol template draft has been created and is being further modified by feedback from the research community. DISCUSSION/SIGNIFICANCE OF IMPACT: Use of the NIH behavioral trial protocol template is expected to enhance the quality of any behavioral study, because the template and supporting materials were developed with the unique aspects of behavioral research in mind.

